# Relationship between starch fine structure and simulated oral processing of cooked *japonica* rice

**DOI:** 10.3389/fnut.2022.1046061

**Published:** 2022-11-03

**Authors:** Guodong Liu, Ruizhi Wang, Shaoqiang Liu, Man Xu, Lunan Guo, Hongcheng Zhang, Haiyan Wei

**Affiliations:** ^1^Jiangsu Co-Innovation Center for Modern Production Technology of Grain Crops, Yangzhou University, Yangzhou, China; ^2^Jiangsu Key Laboratory of Crop Genetics and Physiology/Jiangsu Key Laboratory of Crop Cultivation and Physiology, Agricultural College of Yangzhou University, Yangzhou, China; ^3^Research Institute of Rice Industrial Engineering Technology of Yangzhou University, Yangzhou, China; ^4^School of Food Science and Engineering, Yangzhou University, Yangzhou, China

**Keywords:** *japonica* rice, oral processing, starch fine structure, eating quality, palatability

## Abstract

**Background:**

Simulated oral processing can be used to evaluate the palatability of cooked rice. Previously, we established a simulated oral processing method using a texture analyzer equipped with a multiple extrusion cell probe (TA/MEC). However, the relationship between oral processing and starch fine structure remains unknown.

**Methods:**

In this study, we analyzed the oral processing properties using TA/MEC and characterized the starch fine structure of *japonica* rice by size-exclusion chromatography (SEC) and fluorophore-assisted capillary electrophoresis (FACE). The relationship between starch fine structure and oral processing of cooked *japonica* rice was further investigated.

**Results:**

Cooked rice structure contains fast-breakdown (Type I structure), slow-breakdown (Type II structure) and unbreakable structures (Type III structure). Fast-breakdown and slow-breakdown structure were positively correlated with the content of amylose and shorter amylopectin branches. The content of longer amylopectin branches was positively correlated with the contribution of unbreakable structure.

**Conclusion:**

The results indicated that cooked *japonica* rice varieties with more amylose and shorter amylopectin branches tend to form a harder texture and need more work to break down the fast and slow breakdown structures related to rice kernel fragmentation. Meanwhile, cooked *japonica* rice varieties possess stronger molecular entanglements due to their longer amylopectin branches and contribute more to the breakdown of unbreakable structures. These results can guide breeders to select rice varieties with desirable eating qualities for cultivation.

## Introduction

Rice is a key staple food for human beings and its yield has greatly increased owing to the combination of rice breeding and cultivation technology. However, rice quality, especially eating quality, needs further improvement. The primary consideration of rice customers has shifted from grain yield to grain palatability (1). Theoretically, the palatability of rice can be evaluated by amylose content, gel consistency, and gelatinization, which significantly correlate with the texture formation of cooked rice (2, 3). Researchers usually evaluate the palatability of cooked rice by a human sensory test, but this method is time-consuming and subjective (4, 5).

Oral processing properties, like the force and work during the first bite, chewing, and swallowing, is a crucial procedure for the consumption and palatability of foods (6, 7). Rice is primarily consumed in the form of polished white rice which possesses a whole-grain structure (8), and its oral processing property plays an essential role in consumer acceptability (9, 10). However, the relationship between oral processing and palatability of cooked rice has not been adequately investigated.

Our previous work established a simulated oral processing method by monitoring changes in force and work during mastication using a texture analyzer equipped with a multiple extrusion cell probe (TA/MEC) (11). We found that this method can be used to evaluate the mouthfeel and palatability of cooked rice during mastication (12, 13). In this method, the structure of cooked rice is divided into three types: fast breakdown structure (defined as Type I structure), slow breakdown structure (defined as Type II structure), and unbreakable structure (defined as Type III structure). Based on previous research, the Type I structure is related to the fragmentation of rice kernel, the Type III structure represents a structure that cannot be broken even after much mastication, while the Type II structure lies between these which is related to the fragmentation of rice kernel and the enzymatic degradation of rice matrix (11). The work and proportion of these structures can be used to evaluate rice palatability (11).

Starch consists of two polymers, namely linear amylose and branched amylopectin. Besides, starch is the most abundant component in rice grains. Thus, the structure and content of starch is closely related to the palatability of cooked rice (14). Previous research has confirmed that the texture of cooked rice closely relate to the fine structure of starch, which include the content of amylose, degree of polymerization (DP), and side chain length of amylopectin (15–17). However, the relationship between the oral processing of cooked rice and starch fine structure is still unknown. Thus, this study chose different *japonica* rice varieties as materials and established the relationship between their oral processing properties and starch fine structure. This study can assist researchers in improving rice palatability by using a simulated oral processing method and choosing rice varieties with certain starch fine structures.

## Materials and methods

### Materials

Twelve *japonica* rice varieties were chosen and planted at Shatou town research farm (Yangzhou city, Jiangsu province, China, 32°32′ N, 119°49′ E) from May to November 2019. All rice varieties were planted under the same cultivation conditions for high yield and quality and milled using Xiba LTJM-2099 rice-milling machine (Zhejiang Boliheng Corporation, China). Protease (≥3.5 units/mg solid) and isoamylase (≥10,000,000 units/mg protein) were purchased from Sigma-Aldrich Chemical Co. (St. Louis, MO, USA). Pullulan standards (molecular weight: (Mw) 180 Da ∼ 1.2 × 10^6^ Da) were purchased from Polymer Standards Service (PSS, Mainz, Germany). Simulated saliva (pH 6.8) and low-temperature α-amylase (2,000 U/g solid) were purchased from Ke Lei Biological Technology Co., Ltd. (Shanghai, China) and Shanghai Yuanye Bio-Technology Co., Ltd. (Shanghai, China), respectively. All other chemicals used in this study were of reagent grade.

### Size-exclusion chromatography

The molecular size distributions of whole branched starch (R_*h*_, nm) and debranched starch (Mw, Da) were analyzed using an LC-20AD (Shimadzu, Kyoto, Japan) size-exclusion chromatography (SEC) system equiped with a RID-10A detector, according to the method described by Gilbert (16, 18, 19). Starch was isolated from rice kernels using protease to cleave proteins and anhydrous ethanol to remove lipids. The starch was debranched using isoamylase in order to analyze the chains. All the starch samples (2 mg/mL) were dissolved in DMSO/LiBr solution (0.5%, w/w). Whole starch molecules were separated using a combination of GRAM pre-column, GRAM 30, and GRAM 3000 analytical columns (PSS), while starch chains were separated using a combination of GRAM pre-column, GRAM 100, and GRAM 1000 analytical columns (PSS). The mobile phase was DMSO/LiBr solution (0.5%, w/w) with an elution rate of 0.3 and 0.6 mL/min for branched and debranched starches, respectively. The column oven temperature was maintained at 80 °C. Pullulan standards (PSS) with different Mw(180 Da ∼ 1.2 × 10^6^ Da) were used for calibration and calculation. The data were analyzed using XPS Peak Fit software v. 4.1. After peak fitting, the SEC data were calculated to characterize the molecular size distributions of the branched and debranched starch samples.

### Fluorophore-assisted capillary electrophoresis

The chain length distributions (CLD) of amylopectin were analyzed according to the method described by Gilbert (20). Amylopectin in the starches was firstly debranched using isoamylase, then labeled with 8-aminopyrene-1,3,6-trisulfonic acid. After that, the samples was analyzed using a PA-800 Plus fluorophore-assisted capillary electrophoresis (FACE) system (Beckman Coulter, Brea, CA, USA) with a solid-state laser-induced fluorescence detector and an argon-ion laser as the excitation source.

### Simulated oral processing

The oral processing properties of the cooked rice were measured according to a previous method (11). Milled rice was cooked at a rice/water ratio of 1:1.3 using a rice cooker. The oral processing properties of the cooked rice were measured using a TA.XTplus TA/MEC (Stable Micro System, Surrey, UK). Briefly, cooked rice (30 g) together with simulated saliva (3.6 mL, pH6.8) and low-temperature α-amylase (0.01 g, ezyme activity: 2000 U/g solid) was added to the MEC probe according to the in situ oral processing of human subjects (11). Cyclic compression was conducted 25 times to destroy the structure of the rice kernels. The extrusion distance was set to 93 mm, and both the test and post-test speeds were set as 5 mm/s. The work during each chewing cycle was calculated according to the force versus distance curves and analyzed by fitting to the double-exponential decay function (Eq. (1)):


(1) $$w_{n}=w_{\infty }+w_{1}\,e^{\left(-\frac{n}{n_{1}}\right)}+w_{2}\,e^{\left(-\frac{n}{n_{2}}\right)}$$

where *w*_*n*_ is the work during each compression cycle (n), *w*_1_ and *w*_2_ are the contributions to the loss of energies per cycle with a decay rate given by *n*_1_ and *n*_2_, respectively. Besides, *w*_∞_ is the work per cycle even after an infinite number of compression cycles.

In this method, *w*_*n*_ can be divided into three parts: *w_*Type*__*I*_*, *w_*Type*_ _*II*_*, and *w_*Type*_ _*III*_*, which represent the work per cycle to break down Type I (Eq. (2)), Type II (Eq. (3)), and Type III (Eq. (4)) structures, respectively. In addition, the fractions of *w_*Type*__*I*_* (*f_*Type*__*I*_*), *w_*Type*_ _*II*_* (*f_*Type*_ _*II*_*), and *w_*Type*_ _*III*_* (*f_*Type*_ _*III*_*) during each compression cycle can be calculated according to Eqs. (5), (6), and (7).


(2) $$w_{T\,y\,p\,e\,I}=w_{1}\,e^{\left(-\frac{n}{n_{1}}\right)}$$

(3) $$w_{T\,y\,p\,e\,I\,I}=w_{2}\,e^{\left(-\frac{n}{n_{2}}\right)}$$

(4) $$w_{T\,y\,p\,e\,I\,I\,I}=w_{\infty }$$

(5) $$f_{T\,y\,p\,e\,I}\;(\%)=\frac{100\,w_{T\,y\,p\,e\,I}}{w_{n}}$$

(6) $$f_{T\,y\,p\,e\,I\,I}\;(\%)=\frac{100\,w_{T\,y\,p\,e\,I\,I}}{w_{n}}$$

(7) $$f_{T\,y\,p\,e\,I\,I\,I}\;(\%)=\frac{100\,w_{T\,y\,p\,e\,I\,I\,I}}{w_{n}}$$

During the whole oral processing, the total work to break down Type I (*W_*Type*__*I*_*), Type II (*W_*Type*_ _*II*_*), and Type III (*W_*Type*_ _*III*_*) structures were calculated according to Eq. (8), Eq. (9) and Eq. (10) and their corresponding fractions (*F_*Type*__*I*_*, *F_*Type*_ _*II*_*, *F_*Type*_ _*III*_*) were calculated according to Eq. (11), Eq. (12) and Eq. (13), respectively.


(8) $$W_{T\,y\,p\,e\,I}=\sum_{i=1}^{50}w_{1}\,e^{\left(-\frac{i}{n_{1}}\right)}$$

(9) $$W_{T\,y\,p\,e\,I\,I}=\sum_{i=1}^{50}w_{2}\,e^{\left(-\frac{i}{n_{2}}\right)}$$

(10) $$W_{T\,y\,p\,e\,I\,I\,I}=\sum_{i=1}^{50}w_{\infty }$$

(11) $$F_{T\,y\,p\,e\,I}\;(\%)=\frac{100\,W_{T\,y\,p\,e\,I}}{W_{T\,y\,p\,e\,I}\;+\;W_{T\,y\,p\,e\,I\,I}\;+\;W_{T\,y\,p\,e\,I\,I\,I}}$$

(12) $$F_{T\,y\,p\,e\,I\,I}\;(\%)=\frac{100\,W_{T\,y\,p\,e\,I\,I}}{W_{T\,y\,p\,e\,I}\;+\;W_{T\,y\,p\,e\,I\,I}\;+\;W_{T\,y\,p\,e\,I\,I\,I}}$$

(13) $$F_{T\,y\,p\,e\,I\,I\,I}\;(\%)=\frac{100\,W_{T\,y\,p\,e\,I\,I\,I}}{W_{T\,y\,p\,e\,I}\;+\;W_{T\,y\,p\,e\,I\,I}\;+\;W_{T\,y\,p\,e\,I\,I\,I}}$$

### Statistical analyses

Correlations between the starch fine structure ans the oral processing properties of cooked rice were analyzed using IBM SPSS^®^ AmosTM 19 (SPSS Inc., Chicago, IL, USA). Both Pearson and Spearman rank correlations were analyzed at *p* < 0.05 and *p* < 0.01 for significant and quite significant correlations, respectively.

## Results and discussion

### Starch fine structure of *japonica* rice

#### Molecular size distributions of branched and debranched starches

**FIGURE 1:**
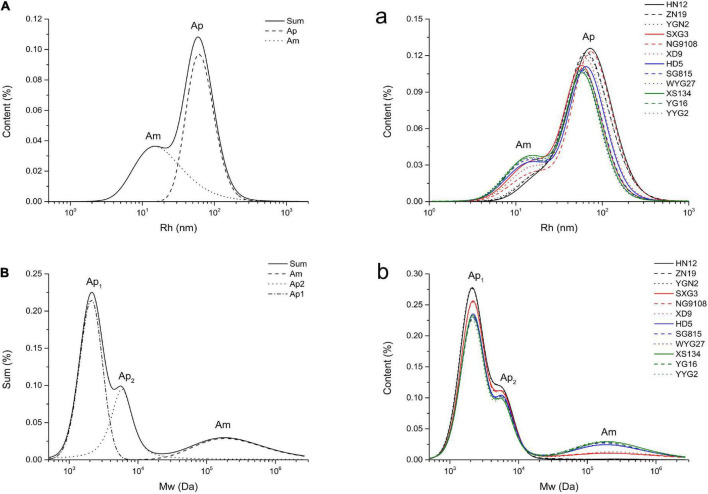
Molecular size distributions of whole branched starches **(A/a**, Rh**)** and debranched starches **(B/b**, Mw**)** extracted from *japonica* rice.

The molecular size distributions of branched starches extracted from different *japonica* rice varieties are shown in Figure 1A. Two peaks were observed in the molecular size distribution curves, which were analyzed using XPS peak fitting software (Figure 1A). The lower and higher peaks around 10 to 20 nm and 60 to 100 nm are associated with amylose and amylopectin, respectively, in rice starch (21). Based on the structural parameters summarized in Table 1, the average R_*h*_ values of the whole rice starch (Rh_*sum*_) ranged from 49.58 to 74.98 nm. As previously mentioned, the SEC method can only semi-quantitatively analyze the whole starch molecular size distribution and cannot calculate amylose content because of the unavoidable shear scission effects and unsatisfactory separation of amylose and amylopectin (22, 23). Thus, branched rice starches were thoroughly debranched and characterized using SEC to analyze their fine structures (Figures 1B,b).

**TABLE 1:** Starch structural parameters of whole branched starch and debranched starch extracted from *japonica* rice.

Samples	Average Rh of branched starches (nm)			Average molecular weight of debranched starches (Da)				Content (%)				
			
	Rh_*Sum*_^1^	Rh_*Ap*_^2^	Rh_*Am*_^3^	MW_*Sum*_^4^	MW_*Ap1*_^5^	MW_*Ap2*_^6^	MW_*Am*_^7^	C_*Ap1*_^8^	C_*Ap2*_^9^	C_*Am*_^10^
HN12	74.98	86.96	41.62	5635	2203	8875	187409	65.67	33.97	0.36
ZN19	73.22	81.40	43.20	7034	2213	9961	202326	64.82	34.09	1.09
YGN2	71.57	82.37	41.58	5595	2172	9423	172848	65.66	33.77	0.57
SXG3	56.50	69.14	40.50	34378	2261	11976	371304	60.35	31.79	7.87
NG9108	73.69	89.19	42.33	32630	2268	13520	365379	60.44	32.20	7.36
XD9	66.26	76.18	49.74	36504	2278	13454	405694	60.58	31.82	7.60
HD5	58.57	77.05	34.48	53607	2272	14535	339170	54.80	31.09	14.11
SG815	55.74	71.58	35.89	56284	2271	14819	318786	53.57	30.58	15.85
WYG27	51.93	66.69	34.98	53425	2276	15299	325448	54.37	31.06	14.58
XS134	52.05	68.85	34.26	61961	2247	14702	344455	54.18	29.44	16.38
YG16	56.43	69.96	37.88	55896	2265	15019	311744	53.83	30.08	16.09
YYG2	49.58	69.24	28.69	63936	2232	15926	332257	52.71	29.83	17.46

^1^Rh_*Sum*_: average radius of the whole rice starch; ^2^Rh_*Ap*_: average radius of amylopectin; ^3^Rh_*Am*_: average radius of amylose; ^4^MW_*Sum*_: molecular weight of the whole debranched rice starch; ^5^MW_*Ap1*_: molecular weight of shorter amylopectin branches; ^6^MW_*Ap2*_: molecular weight of longer amylopectin branches; ^7^MW_*Am*_: molecular weight of amylose; ^8^C_*Ap1*_: content of shorter amylopectin branches; ^9^C_*Ap2*_: content of longer amylopectin branches; ^10^C_*Am*_: content of amylose.

After debranching, the α-1,6-glycosidic bonds at branching points in amylopectin were cleaved, leading to the generation of short linear starch chains. The Mw distribution curves of the debranched rice starches show three peaks, including two larger peaks of amylopectin branches and one smaller peak of amylose branches. The peak at approximately 2 × 10^3^ Da (Ap_1_) represents shorter amylopectin branches that are confined to a single lamella, whereas the peak at approximately 5 × 10^3^ to 6 × 10^3^ Da (Ap_2_) is associated with longer amylopectin branches that span two or more lamellae (16, 24, 25). The peak at approximately 2 × 10^5^ Da (Am) represents amylose (26). The Mw and content of the different starch branches were analyzed by calculating the area under the curve of each peak after peak fitting (Figure 1B); all the structural parameters are shown in Table 1.

The amylose content (C_*Am*_) of the *japonica* rice varieties used in this study ranged from 0.36% to 17.46%. Waxy *japonica* rice starches, including HN12, ZN19, and YGN2, showed a very limited amount of Am (C_*Am*_ < 2%) and shorter Am and Ap_2_ chains. Meanwhile, the C_*Am*_ values of SXG3, NG9108, and XD9 was approximately 7% to 8%, and these rice varieties have been defined as soft *japonica* rice. Soft *japonica* rice starches had longer Am chains, whereas other common *japonica* rice starches (C_*Am*_: 14.11% to 17.46%) had fewer Ap_1_ and Ap_2_ chains and much longer Ap_2_ chains. The *japonica* rice varieties used in this study showed different fine starch structures, including Mw and chain length of both amylose and amylopectin.

#### Chain-length distributions of amylopectin

Compared to SEC, FACE can separate and characterize the distributions of individual chains (27). FACE can measure the content of starch chains with DP values ranging from 6 to 100; these starch chains are defined as amylopectin (Ap) chains (26). Theoretically, the CLD of amylopectin can be fractionated into five parts, namely the A Chain (6 ≤ DP ≤ 12), B_1_ Chain (13 ≤ DP ≤ 24), B_2_ Chain (25 ≤ DP ≤ 36), B_3_ Chain (37 ≤ DP ≤ 65), and C Chain (DP ≥ 66) (27, 28). The CLD of amylopectin measured using FACE are shown in Table 2.

**TABLE 2:** Chain length distribution (CLD) analysis of debranched rice starches extracted from *japonica* rice.

Samples	Starch chain length distribution (%)					Average CL* (DP)
	A Chain (6 ≤ DP ≤ 12)	B_1_ Chain (13 ≤ DP ≤ 24)	B_2_ Chain (25 ≤ DP ≤ 36)	B_3_ Chain (37 ≤ DP ≤ 65)	C Chain (DP ≥ 66)	
HN12	28.31	46.61	10.65	12.71	1.72	21.33
ZN19	28.93	46.49	10.66	12.32	1.60	21.08
YGN2	28.65	46.34	10.57	12.75	1.68	21.27
SXG3	28.24	47.07	10.69	12.36	1.63	21.16
NG9108	27.96	46.86	10.70	12.79	1.69	21.35
XD9	28.44	46.87	10.64	12.46	1.59	21.13
HD5	28.78	47.07	10.43	12.11	1.62	20.98
SG815	29.24	47.43	10.47	11.50	1.36	20.59
WYG27	29.02	47.64	10.41	11.63	1.30	20.60
XS134	29.27	47.33	10.39	11.55	1.46	20.66
YG16	28.70	47.39	10.33	12.01	1.56	20.91
YYG2	28.64	47.49	10.54	11.93	1.39	20.79

*CL: average chain length of amylopectin branches.

The results indicated that amylopectin extracted from waxy and semi-waxy *japonica* rice varieties had longer chain lengths and more B_3_ and C chains than common rice varieties. All the *japonica* rice amylopectins had higher amounts of the B_1_ Chain (46.34 to 47.64%) than other fractionated parts. In addition, the amount of the A Chain fraction in all samples was similar, which is related to the determination of starch crystalline polymorphs (27).

### Simulated oral processing of cooked *japonica* rice

**FIGURE 2:**
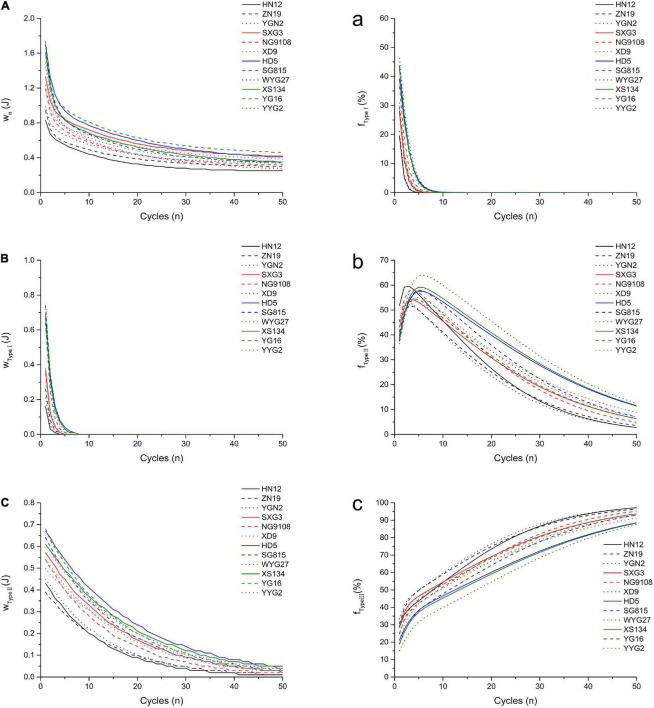
Simulated oral processing of cooked *japonica* rice. The fitted work of three type structures during each extrusion cycle **(A)** Type I; **(B)** Type II; **(C)** Type III. The fraction of the work contributed to the breakdown of different structures per extrusion cycle **(a)** Type I; **(b)** Type II; **(c)** Type III.

TA/MEC was used to measure the oral processing properties of cooked *japonica* rice (11). The work and force applied to the cooked rice during each chewing cycle were used to characterize their oral processing properties. The results were shown in Figure 2 and Table 3.

**TABLE 3:** Parameters from the fitting model of simulated oral processing of cooked *japonica* rice.

	Double-exponential decay												
Samples	function parameters					*W_1_,^3^*	*W_Type__I_^4^*	*W_Type_ _II_^5^*	*W_Type_ _III_^6^*	*W_Total_^7^*	*F_Type__I_^8^*	*F_Type_ _II_^9^*	*F_Type_ _III_^10^*
	*w_∞_^1^*	*w_1_^2^*	*n*_1_	*w_2_^2^*	*n*_2_	(J)	(J)	(J)	(J)	(J)	(%)	(%)	(%)
HN12	0.24	0.77	0.64	0.47	11.82	0.83	0.20	5.25	12.00	17.45	1.17	30.06	68.77
ZN19	0.29	0.87	0.84	0.42	13.64	0.94	0.38	5.38	14.50	20.26	1.88	26.55	71.57
YGN2	0.33	0.95	0.66	0.49	12.69	0.99	0.27	5.86	16.50	22.63	1.18	25.90	72.92
SXG3	0.39	1.14	0.88	0.61	15.91	1.33	0.54	9.00	19.50	29.04	1.86	30.99	67.16
NG9108	0.30	1.03	0.93	0.58	13.74	1.19	0.53	7.48	15.00	23.01	2.32	32.51	65.18
XD9	0.35	1.09	0.95	0.53	18.16	1.23	0.58	8.77	17.50	26.85	2.18	32.65	65.18
HD5	0.36	1.36	1.40	0.71	18.33	1.70	1.30	11.83	18.00	31.14	4.19	38.01	57.81
SG815	0.32	1.62	1.28	0.68	14.98	1.70	1.37	9.50	16.00	26.87	5.09	35.36	59.55
WYG27	0.24	1.57	1.32	0.65	16.95	1.59	1.39	10.14	12.00	23.52	5.89	43.09	51.02
XS134	0.31	1.5	1.28	0.65	18.03	1.61	1.27	10.69	15.50	27.45	4.61	38.92	56.46
YG16	0.43	1.68	1.03	0.72	15.59	1.74	1.02	10.43	21.50	32.95	3.11	31.65	65.24
YYG2	0.32	1.45	1.20	0.58	15.65	1.49	1.11	8.43	16.00	25.54	4.36	33.00	62.64

^1^*w_n_*: the work during each compression cycle (n); ^2^*w_1_*, *w_2_*: the contributions to the loss of energies per cycle with a decay rate given by *n_1_* and *n_2_*, respectively. ^3^*W_1_*: the work done during the first bite; ^4^*W_Type__I_*: the total work to break down Type I structure; ^5^*W_Type_ _II_*: the total work to break down *W_Type_ _II_* structure; ^6^*W_Type_ _III_*: the total work to break down *W_Type_ _III_* structure; ^7^*W_Total_*: the total work during the whole oral processing; ^8^*F_Type__I_*: the fraction of *W_Type__I_*; ^9^*F_Type_ _II_*: the fraction of *W_Type_ _II_*; ^10^*F_Type_ _III_*: the fraction of *W_Type_ _III_*.

The results showed that *w_*Type*__*I*_* and its fraction (*f_*Type*__*I*_*) decreased rapidly with the compression cycles, and this can be used to analyze and quantify the fast breakdown structure (Type I) in cooked japonica rice. However, *w_*Type*_ _*II*_* decreased slowly with the compression cycles, which represents the slow breakdown structure (Type II) in cooked rice. The fraction of *w_*Type*_ _*II*_* (*f_*Type*_ _*II*_*) first increased and then decreased, while the fraction of *w_*Type*_ _*III*_* (*f_*Type*_ _*III*_*) increased gradually with the number of compression cycles. These results indicate that the Type I structure plays a more important role in the initial stage of chewing cooked rice, especially within five compression cycles. After approximately five compression cycles, more work per chewing cycle was required to break down the Type II structure. However, the type III structure gradually became the dominant factor affecting the oral processing properties of cooked rice from approximately the tenth compression cycle to the end.

Different *japonica* rice varieties showed a significant variance in oral processing properties. Based on the double-exponential decay function parameters, common *japonica* rice varieties (HD5, SG815, YG16, etc.) possess higher *w*_1_, *w_2_*, and *w*_∞_ values, indicating that these rice varieties require more work to break down Type I, Type II, and Type III structures during oral processing than waxy *japonica* rice (HN12, ZN19, and YGN2) and soft *japonica* rice varieties (SXG3, NG9108, and XD9). Meanwhile, *japonica* rice varieties with larger *n*_1_ and *n*_2_ values require more extrusions to break down the Type I and Type II structures. *W*_1_ is the work done during the first bite, and its value closely relates to the hardness of cooked rice (6). These results indicated that the hardness of cooked common *japonica* rice was higher than that of other *japonica* rice varieties. During entire oral processing, more work is required to break down the three structure types for common *japonica* rice varieties, while the contributions of the different structure types to the oral processing properties perform differently. Common *japonica* rice varieties possess higher *F_*Type*__*I*_* values and lower *F_*Type*_ _*III*_* values than waxy and soft *japonica* rice varieties. These results indicate that the Type I structure contributes more to the oral processing properties of common *japonica* rice varieties, whereas the Type III structure in cooked waxy *japonica* rice varieties plays a more important role in the formation of oral processing properties.

### Correlations between starch fine structure and simulated oral processing of *japonica* rice

The relationships between starch fine structure and simulated oral processing of *japonica* rice varieties were further investigated by Pearson and Spearman rank correlation analysis, and the results are summarized in Table 4. Pearson correlation can analyze the linear correlations, while Spearman rank correlation can reflect non-linear correlations. Both Pearson and Spearman correlation tests show that the double-exponential decay function parameters, including *w*_1_, *n*_1_, and *w*_2_, were significantly and negatively correlated with Rh_*sum*_. *W*_1_, *W_*Type*__*I*_*, *W_*Type*_ _*II*_*, *F_*Type*__*I*_*, and *F_*Type*_ _*II*_* showed the same relationship with Rh_*sum*_. However, *w*_∞_ and *W_*Type*_ _*III*_* showed no significant relationship with Rh_*sum*_, but *F_*Type*_ _*III*_* significantly and positively related to Rh_*sum*_. These results indicate that *japonica* rice varieties with smaller starch molecular sizes tend to form harder textures and need more work to break down the Type I and Type II structures. Simultaneously, the Type I and Type II structures in *japonica* rice varieties with smaller starch molecular sizes contribute more to the oral processing properties, whereas the Type III structure contributes less. In *japonica* rice starches, the Rh_*sum*_ of amylopectin is significantly larger than that of amylose. Thus, *japonica* rice varieties with larger Rh_*sum*_s possess less amylose.

**TABLE 4:** Correlation coefficients between starch fine structure and simulated oral processing of cooked *japonica* rice.

	Average Rh of			Average molecular weight of							Starch Chain Length					Average
Pearson	branched starches (nm)			debranched starches (Da)				Content (%)			Distribution (%)					CL
	Rh_*Sum*_	Rh_*Ap*_	Rh_*Am*_	MW_*Sum*_	MW_*Ap1*_	MW_*Ap2*_	MW_*Am*_	C_*Ap1*_	C_*Ap2*_	C_*Am*_	A	B_1_	B_2_	B_3_	C	(DP)
*w*_∞_	–0.275	–0.322	0.037	0.301	0.286	0.263	0.147	–0.267	–0.366	0.293	–0.118	0.128	–0.220	–0.011	0.177	0.043
*w*_1_	−0.884**	−0.812**	−0.675*	0.921**	0.600*	0.889**	0.280	−0.954**	−0.909**	0.951**	0.586*	0.918**	−0.850**	−0.868**	−0.785**	−0.866**
*n*_1_	−0.798**	−0.652*	−0.682*	0.894**	0.674*	0.879**	0.377	−0.909**	−0.815**	0.893**	0.598*	0.822**	−0.693*	−0.839**	−0.748**	−0.833**
*w*_2_	−0.750**	−0.623*	–0.562	0.855**	0.714**	0.810**	0.450	−0.866**	−0.831**	0.865**	0.325	0.798**	−0.753**	−0.652*	–0.472	−0.611*
*n*_2_	−0.681*	−0.662*	–0.243	0.741**	0.716**	0.720**	0.564	−0.672*	−0.709**	0.687*	0.356	0.598*	–0.491	–0.573	–0.453	–0.551
*W*_1_	−0.863**	−0.759**	−0.641*	0.941**	0.703*	0.902**	0.387	−0.957**	−0.912**	0.954**	0.508	0.876**	−0.807**	−0.808**	−0.664*	−0.784**
*W_*Type*__*I*_*	−0.857**	−0.732**	−0.735**	0.917**	0.624*	0.885**	0.314	−0.949**	−0.859**	0.935**	0.665*	0.888**	−0.805**	−0.907**	−0.817**	−0.905**
*W_*Type*_ _*II*_*	−0.795**	−0.702*	–0.489	0.883**	0.772**	0.839**	0.531	−0.862**	−0.852**	0.866**	0.391	0.779**	−0.726**	−0.688*	–0.504	−0.649*
*W_*Type*_ _*III*_*	–0.275	–0.322	0.037	0.301	0.286	0.263	0.147	–0.267	–0.366	0.293	–0.118	0.128	–0.220	–0.011	0.177	0.043
*W*_*Total*_	−0.646*	−0.617*	–0.291	0.712**	0.618*	0.663*	0.383	−0.683*	−0.731**	0.700*	0.185	0.550	–0.573	–0.435	–0.219	–0.382
*F_*Type*__*I*_*	−0.813**	−0.694*	−0.732**	0.852**	0.568	0.843**	0.272	−0.890**	−0.779**	0.870**	0.677*	0.879**	−0.723**	−0.908**	−0.898**	−0.927**
*F_*Type*_ _*II*_*	−0.683*	–0.563	–0.521	0.758**	0.683*	0.746**	0.530	−0.751**	−0.683*	0.740**	0.443	0.786**	−0.611*	−0.718**	−0.675*	−0.716**
*F_*Type*_ _*III*_*	0.733**	0.609*	0.587*	−0.800**	−0.670*	−0.788**	–0.477	0.804**	0.723**	−0.791**	–0.513	−0.828**	0.654*	0.783**	0.747**	0.786**
**Spearman**	**Rh_*Sum*_**	**Rh_*Ap*_**	**Rh_*Am*_**	**MW_*Sum*_**	**MW_*Ap1*_**	**MW_*Ap2*_**	**MW_*Am*_**	**C_*Ap1*_**	**C_*Ap2*_**	**C_*Am*_**	**A**	**B_1_**	**B_2_**	**B_3_**	**C**	**CL**

*w*_∞_	–0.172	–0.161	–0.102	0.242	0.204	0.109	0.175	–0.267	–0.295	0.281	–0.158	0.081	–0.200	0.007	0.021	0
*w*_1_	−0.839**	−0.734**	−0.671*	0.832**	0.503	0.874**	–0.035	−0.902**	−0.888**	0.881**	0.538	0.886**	−0.783**	−0.811**	−0.804**	−0.825**
*n*_1_	−0.732**	−0.595*	−0.718**	0.795**	0.680*	0.809**	0.172	−0.757**	−0.753**	0.764**	0.599*	0.786**	−0.697*	−0.753**	−0.729**	−0.785**
*w*_2_	−0.607*	–0.530	−0.635*	0.695*	0.554	0.698*	0.081	−0.751**	−0.761**	0.719**	0.407	0.714**	−0.712**	−0.621*	–0.502	−0.607*
*n*_2_	–0.545	−0.608*	–0.427	0.594*	0.713**	0.490	0.594*	–0.448	–0.566	0.566	0.259	0.518	–0.434	–0.427	–0.462	–0.448
*W*_1_	−0.718**	−0.602*	−0.680*	0.813**	0.536	0.778**	0.028	−0.841**	−0.841**	0.827**	0.522	0.767**	−0.788**	−0.739**	−0.651*	−0.739**
*W_*Type*__*I*_*	−0.818**	−0.706*	−0.706*	0.804**	0.678*	0.839**	0.112	−0.804**	−0.776**	0.790**	0.636*	0.872**	−0.692*	−0.839**	−0.839**	−0.874**
*W_*Type*_ _*II*_*	−0.643*	−0.643*	−0.643*	0.706*	0.601*	0.636*	0.224	−0.664*	−0.762**	0.720**	0.524	0.655*	−0.748**	−0.650*	–0.545	−0.643*
*W_*Type*_ _*III*_*	–0.172	–0.161	–0.102	0.242	0.204	0.109	0.175	–0.267	–0.295	0.281	–0.158	0.081	–0.200	0.007	0.021	0
*W*_*Total*_	–0.531	–0.545	–0.483	0.657*	0.490	0.510	0.308	−0.643*	−0.699*	0.678*	0.224	0.536	–0.545	–0.476	–0.364	–0.441
*F_*Type*__*I*_*	−0.804**	−0.636*	−0.692*	0.818**	0.566	0.895**	0.028	−0.832**	−0.783**	0.811**	0.664*	0.858**	−0.664*	−0.839**	−0.867**	−0.874**
*F_*Type*_ _*II*_*	−0.699*	−0.615*	−0.664*	0.755**	0.678*	0.755**	0.350	−0.671*	−0.727**	0.706*	0.517	0.785**	−0.594*	−0.706*	−0.699*	−0.720**
*F_*Type*_ _*III*_*	0.690*	0.599*	0.673*	−0.750**	−0.666*	−0.760**	–0.343	0.676*	0.725**	−0.704*	–0.508	−0.782**	0.581*	0.697*	0.683*	0.708*

*Correlations are significant at *p* < 0.05. **Correlations are significant at *p* < 0.01.

The relationship between amylose content and oral processing properties of *japonica* rice varieties suggests that *japonica* rice varieties with higher amylose content possess higher *w*_1_, *n*_1_, *w_2_*, and *n*_2_ values, have a harder texture, and need more work to break the Type I and Type II structures down. In addition, the *F_*Type*__*I*_* and *F_*Type*_ _*II*_* values were significantly and positively correlated with the amylose content of *japonica* rice starches, while the *F_*Type*_ _*III*_* values showed a significant and negative correlation with the amylose content of *japonica* rice starches. These results indicate that Type I and Type II structures in *japonica* rice varieties with higher amylose content contribute more to the oral processing properties, while the Type III structure contributes less.

The relationship between the CLD of amylopectin parameters and the oral processing properties of *japonica* rice varieties was further analyzed. Theoretically, A- and B_1_-chains represent shorter branches on the outer side of amylopectin, while B_2_-, B_3_-, and C-chains represent longer branches on the inner side (27). The content of B_1_-chains with the highest amounts of amylopectin fractions significantly and positively correlated with *w*_1_, *n*_1_, *w*_2_, *n*_2_,*W_1_*, *W_*Type*__*I*_*, *W_*Type*_ _*II*_*, *F_*Type*__*I*_*, and *F_*Type*_ _*II*_*, and significantly and negatively correlated with *F_*Type*_ _*III*_*. However, the content of longer side chains in amylopectin, including B_2_-, B_3_-, and C-chains, and the average chain length of amylopectin, showed an opposite correlation with the oral processing parameters of cooked *japonica* rice. These results indicate that *japonica* rice varieties with more short side chains (including A- and B_1_-chains) tend to form a harder texture and need more work to break the Type I and Type II structures down during the whole oral processing of cooked *japonica* rice.

In summary, amylose content is the most critical factor affecting the oral processing properties of cooked *japonica* rice. Cooked *japonica* rice varieties with higher amylose content contain more fast breakdown structure and slow breakdown structure, and both contribute more to oral processing properties. In contrast, cooked *japonica* rice varieties with longer side chains possess fewer fast breakdown structure and slow breakdown structure, while the role of the unbreakable structure is more important in forming oral processing properties.

## Conclusion

This study provides a new perspective on the relationship between starch fine structures and oral processing properties of cooked *japonica* rice. The oral processing properties of cooked *japonica* rice can be determined by evaluating the amount of work contributed to the breakdown of different structures and their corresponding fractions. This study found, for the first time, that amylose content together with shorter branches in amylopectin significantly positively correlated with the amount of work contributed to the fast breakdown structure and slow breakdown structure, as well as their corresponding fractions during oral processing of cooked *japonica* rice. Furthermore, cooked *japonica* rice varieties with longer branches in amylopectin contained fewer fast breakdown structure and slow breakdown structure, while the contribution of the unbreakable structure to oral processing became more important.

We confirmed the fast breakdown structure is controlled by the fragmentation of rice kernel; the unbreakable structure is closely related to the undigested substance, which is controlled by the molecular entanglements among rice components during oral processing, while the slow breakdown structure is mainly controlled by the combination of rice kernel fragmentation and enzymatic degradation (11). Cooked *japonica* rice varieties with more amylose and shorter branches in amylopectin tend to form a harder texture, and more work is needed to contribute to the rice kernel fragmentation relating to the fast breakdown structure and slow breakdown structure. Meanwhile, cooked *japonica* rice varieties with longer branches of amylopectin possess stronger molecular entanglements and contribute more to the breakdown of the unbreakable structure. In summary, this study, for the first time, established the relationship between the oral processing properties of cooked *japonica* rice and its fine starch structure. This research offers a useful method to evaluate rice palatability and offers rice breeders new insights into selecting rice varieties with desirable oral processing properties with the required starch fine structure for cultivation.

## Data availability statement

The original contributions presented in this study are included in the article/supplementary material, further inquiries can be directed to the corresponding author.

## Author contributions

GL: methodology, investigation, data curation, and writing – original draft. RW, SL, and MX: methodology and investigation. LG and HZ: writing – review editing. HW: funding acquisition and writing – review editing. All authors contributed to the article and approved the submitted version.

## Abbreviations

SEC: size-exclusion chromatography
FACE: fluorophore-assisted capillary electrophoresis
Mw: molecular weight
CLD: chain length distributions
DP: degree of polymerization
C_*Am*_: amylose content
Ap: amylopectin
TA/MEC: a texture analyzer equipped with a multiple extrusion cell probe
Type I structure: fast breakdown structure
Type II structure: slow breakdown structure
Type III structure: unbreakable structure.
